# Editorial: Non-biomedical perspectives on pain and its prevention and management

**DOI:** 10.3389/fpain.2024.1404074

**Published:** 2024-05-30

**Authors:** Mark I. Johnson, Antonio Bonacaro, Emmanouil Georgiadis, James Woodall

**Affiliations:** ^1^ Centre for Pain Research, School of Health, Leeds Beckett University, Leeds, United Kingdom; ^2^ Department of Medicine and Surgery, School of Nursing, University of Parma, Parma, Italy; ^3^ School of Social Sciences and Humanities, University of Suffolk, Ipswich, United Kingdom

**Keywords:** pain, ecology, salutogenesis, art, language, metaphor, history, psychosocial

**Editorial on the Research Topic** Non-biomedical perspectives on pain and its prevention and management

## Introduction

Overreliance on the biomedical paradigm has contributed, in part, to overuse of surgery and long-term drug medication with harmful physical, psychological, social, and economic consequences. Research is dominated by a tissue-centric biomedical view of pain at the expense of a holistic first-person *experience* of living with pain in communities of people habiting modern-world settings. Pain practice seems overly consumed with the burden of pain at an individual level (patient-centred pain management) and has neglected exploration of societal level (community-centred) or environmental level (ecologically-centred) solutions.

This Research Topic acknowledges that the biomedical paradigm does not provide a complete understanding of pain by focussing attention upstream towards the role of the environment in fashioning the experience and impact of pain on health. Research methodologies from non-biomedical disciplines can explore social, cultural, economic, political, and environmental conditions that influence the living experience of pain in the modern era. Investigating the phenomenon of pain using socio-ecological frameworks provide opportunities to shift perspectives and open-up new avenues for exploration, including innovative strategies to reduce the burden of pain on society.

The purpose of this Research Topic is to broaden and deepen the conceptual understanding of pain in the modern era by showcasing contributions from non-biomedical disciplines. This includes exploration of the concept of painogenic lifestyles and environments, and non-medical strategies targeting living well with, and recovery from, pain at individual, community, or population levels. Our desire is to catalyse scholarly conversation about the interplay between individuals, society, and ecosystems to gain a better understanding of the phenomenon of pain and to inform future healthcare research, practice, and policy.

The Research Topic is deliberately broad in scope to encourage cross-fertilisation of scholarly disciplines from the humanities and the sciences, e.g., social, natural, formal, and applied. We encouraged articles that offered novel perspectives and invited contributions from Anthropology, Behavioural sciences, Ecology, Evolution, Health promotion, History, Politics, Philosophy, Sociology, Socio-economics, Spirituality, the Arts, and Theology. We accepted seventeen articles, both theoretical and empirical, that cover topics not normally visible in conventional pain science literature including ecology, language, salutogenesis, art, emotional memory, and temporality. Contributions offer viewpoints that curiously and critically explore biomedical dogma to provide a broader and deeper understanding of pain and its persistence within the complex socio-ecological milieu of modern life.

## Overview of contributions

### A socio-ecological model for pain

**Figure 1:**
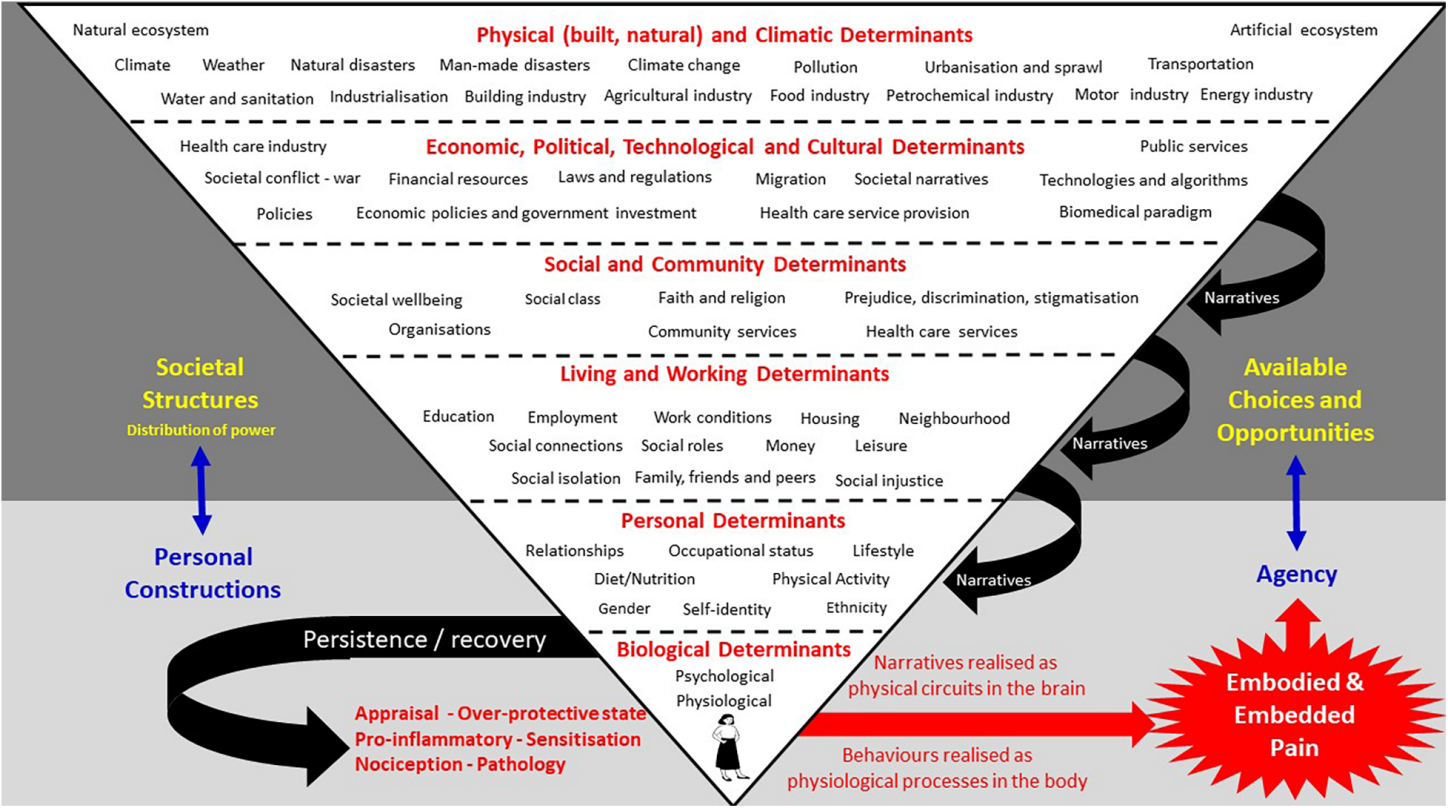
See Johnson and Woodall for an explanation of their socio-ecological model of pain.

Many of the perspectives offered by the contributions are encapsulated in a socio-ecological model of pain presented in the article by Johnson and Woodall and reproduced here in Figure 1.

Johnson and Woodall contend that viewing pain through an evolutionary mismatch lens can reveal insidious “upstream” forces that create painogenic environments (1) and structural barriers to hinder recovery from episodes of pain, i.e., making pain “sticky” [*q.v.* Borsook et al. (2)]. Johnson and Woodall’s socio-ecological model reveals how the biomedically dominant explanatory language of pain bonds upstream forces into a cohesive narrative that primes society about the meaning, prevention, treatment, and management of pain; a type of “social glue”. The contribution by Paley et al. that appraises critiques of the writings of people living with pain during the mid (high) to late Medieval Period (c. 1,000–1,500 AD) reveals how pain was a “shared experience”. Paley et al. argue that sharing of personal stories is a fundamental human attribute to foster social cohesion, and that the dominant biomedical narrative of modern living may be preventing people from connecting with a sense of self and their social world.

### Pain and language

The contribution by Van Rysewyk offers perspective on the role of language about pain, motivated by the 20th century Austrian philosopher Wittgenstein (3). Van Rysewyk reveals how the use of the word “pain” is “…linked to participation in a social milieu where specific rules are learnt for the regulation of concepts” and that “pain is not merely a “raw feel”…” but rather a social construct which is learnt and refined throughout life. Consequently, disagreements between health care professionals about the nature (diagnosis) of a person's pain should be recognized as the “indefiniteness” of pain.

The contribution by Johnson et al. explores pain through the perspective of linguistic relativity, as described by Lakoff and Johnson in the 1980s (4). Johnson et al. describe how metaphor is a tool to shape conceptual understanding of phenomena, such as pain, and that the tissue-centric, neuromechanistic explanatory model of pain creates fallacies and misnomers (5) and an unhealthy focus on biomedical research (6). Moreover, Johnson et al. contend that damage-loaded warmongering pain metaphor shapes a person's lived reality to the detriment of their health and well-being, ultimately making pain sticky. Johnson et al. argue that to move people towards a positive life with or without pain, we “literally” need to change our metaphors and narrative to align with the principles of salutogenesis, i.e., fostering health.

### Pain and salutogenesis

Various contributions explore pain through a salutogenic lens. Salutogenesis is a whole-person approach to grow health, grounded in a person's unique life story and current situation, encompassing physical, mental, social, and spiritual aspects that are meaningful to the individual (7–9). Johnson and Woodall suggest that healthy settings approaches based on the principles of salutogenesis are likely to alleviate painogenicity and the stickiness of pain. Paley and Johnson offer perspective on salutogenic approaches and how mindfulness interventions can be integrated into daily living to improve a person's comprehension, meaningfulness, and manageability of living well in the modern world, i.e., a sense of coherence. Georgiadis and Johnson position positive psychology within a whole-person salutogenic approach to care, and they discuss how incorporation of personal narration could foster an agentic form of positive psychology, conferring synergistic benefits. Georgiadis and Johnson discuss how personal narration combined with positive psychology could lead to advances in policy and professional practice.

Johnson et al. draw upon the principles of salutogenesis in a contribution offering perspective for community-based system change for people living with persistent pain within the context of pain services in the UK. Johnson et al. offer insights from the development and delivery of an innovative pain service called “Rethinking Pain’ that is voluntary and community sector-led rather than medical or therapy-led. The Rethinking Pain service utilises health coaches, social prescribers and link workers who proactively engage with culturally-diverse communities of people who experience the biggest health inequalities, reconnecting them with community-support, including engagement with art.

### Pain and art

A contribution by Koebner et al. explores the role of museums and artists in the effort to reduce the burden of persistent pain, drawing on perspectives and insights from the Analgesic Museum conference. Three domains of interest emerged: exhibition development, arts experiences and practices, and research and creative scholarship. Koebner et al. advocate opportunities for individuals to author their experience of pain and to engage in dialogue about those experiences. This was the purpose of Unmasking Pain, an artist-led project that explored creative approaches to telling stories of life with pain, discussed in the contribution by Johnson et al. People living with persistent pain described Unmasking Pain as “a new set of rules”, providing opportunities for “explorative joy despite pain”. This contrasts with clinical encounters and reveals the potential of art to facilitate expression of complex inner experiences and personal stories to help people make-sense of themselves, shifting them from “I can't do, I am not willing to do it”, to “Perhaps I can, I'll give it a go, I enjoyed”. Engagement with Unmasking Pain also freed-up thinking for pain specialists allowing conceptual thought beyond the biopsychosocial model of pain.

### Pain and emotional memory images

A critical aspect of Johnson and Woodall’s model of pain is that socio-ecological events affect the structure and function of cells, tissue, organs, and systems, i.e., bioplasticity—the ability of bodily structures and processes to adapt (10), including neuroplasticity that reshapes connectivity associated with learning and memory. Several contributions from Hudson and Johnson explore pain and its stickiness from the perspective of non-conscious emotional memory images (EMIs). Hudson and Johnson introduce the notion of EMIs as “Trauma induced, non-conscious, contiguously formed multimodal mental imagery, which triggers an amnesic, anachronistic, stress response within a split-second.” (4) p.1. They contend that encounters in daily living re-trigger EMIs which in turn activate the hypothalamic-pituitary-adrenal (HPA) axis amplifying neural input that may contribute to a debilitating state of psychophysiological dis-ease associated with threat, fear, anxiety, and intractable pain [*q.v.* (11, 12)]. Hudson and Johnson suggest that activities of daily living trigger EMIs and “outdated” stress responses, placing the person in a perpetual state of “alarm”, and they offer some case vignettes to demonstrate how a therapeutic approach, which they call Split-Second Unlearning, may “clear” EMIs to “unblock” the “stickiness” of pain. Hudson and Johnson extend their exploration of EMIs in a contribution that draws upon clinical cases and existing literature to explore how a dysfunctional paternalistic family system, often characterised by authoritarian dynamics, emotional neglect, and abuse, is a fertile ground for the creation of EMIs, potentially making pain sticky.

### Pain and temporality

Hudson and Johnson further explore how EMIs may make pain sticky through the perspective of temporal language. Hudson and Johnson introduce a framework, called PAIN (Past Adversity Influencing Now), comprising notions of Past Perfect, Past Imperfect, Present, Future Imperfect and Future Perfect. Hudson and Johnson explain how the PAIN framework may be used to guide individuals towards a more positive future (Future Perfect) with or without pain. A contribution by Agarwal explores the management of persistent pain through the perspective of temporality, the subjective perception of the flow of chronological time. Agarwal reveals, through an ontologically grounded thematic exploration of Ayurvedic protocols used by physicians from India, that temporality is conceptualized as spatiotemporal present moment awareness and embodied time. The findings provide evidence that more consideration should be given to spatiotemporality as an organizing principle in the management and conceptualisation of persistent pain.

### Psychosocial perspectives

Various contributions advocate the need for greater focus on psychosocial perspectives to support people living with pain. An analysis of data from the 2019 Global Burden of Disease study (13) by Rajkumar provides evidence of the importance of cross-cultural variations in the occurrence of common forms of chronic musculoskeletal pain. Rajkumar reports that the prevalence of chronic neck pain was inversely correlated with Uncertainty Avoidance and the prevalence of chronic low back pain was inversely correlated with the cultural dimensions of Power Distance and Collectivism. Moretti et al. provide evidence that medical curricula of highly ranked universities worldwide are biophysically-focused at the expense of the needs and expectations of patients. Moretti et al. contend that the role of men in biomedical science has been negatively impacting the delivery of high-quality and personalized medical care to women and they offer an innovative education intervention to limit the effects of gender bias on future medical practitioners.

Li and Hapidou report a multidimensional analysis of variables affecting outcome to psychosocial treatments that revealed two groupings (i) anxiety, depression, catastrophizing, somatic symptoms, pain intensity and pre-contemplation, and (ii) contemplation, action, maintenance, activity engagement and pain willingness; these groupings resonate with Jensen et al.'s bivalent Behavioral Inhibition System—Behavioral Activation System (BIS-BAS) model (14). Monaco et al. offer perspective on combining digital technologies such as social media, open data, and Artificial Intelligence to create virtual communities that empower and support patients, the public and practitioners. Monaco et al. conclude that innovative non-biomedical approaches will emerge to improve the understanding of pain and its prevention and management.

## Potential impact

Contributions in this Research Topic confirm the value of broadening the lens through which the persistence (stickiness) of pain is studied. The contributions weave together a variety of perspectives that situate pain at the intersect of tissue and environment to reveal avenues of exploration aligned with pain *experience*. In doing so, the contributions transcend the orthodox tissue-centric biopsychosocial way of thinking to reveal opportunities for scholarship, research, clinical practice, and society that go beyond mainstream pain science. Contributions in this Research Topic demonstrate the power of non-biomedical perspectives to inform whole-person centred approaches, such as contemplative practices and the performing arts, to enable people to curiously explore the relationship between their pain and their living experience. Shifting the focus from pain perception to pain perspective opens a vista of interconnectedness between bodymind, “spirit”, community, and environment; and the realisation of greater need for community-based pain support. We hope that this eBook inspires pain scholars, researchers, and health care practitioners to investigate more thoroughly the complex milieu in which individuals, communities, and populations experience pain, to develop an ecology of pain grounded in a more constructive and meaningful societal narrative.
